# *Lactococcus* Species Central Line-Associated Bloodstream Infection in Pediatrics: A Case Series

**DOI:** 10.3389/fmed.2022.802493

**Published:** 2022-02-03

**Authors:** Sarah E. Firmani, Holly D. Maples, Archana Balamohan

**Affiliations:** ^1^Department of Pharmacy Practice, College of Pharmacy, University of Arkansas for Medical Sciences, Little Rock, AR, United States; ^2^Arkansas Children's Hospital, Little Rock, AR, United States; ^3^Department of Pediatrics, Section of Pediatric Infectious Diseases, University of Arkansas for Medical Sciences, Little Rock, AR, United States

**Keywords:** *Lactococcus*, central line-associated bloodstream infection (CLABSI), bacteremia, pediatric infection, antimicrobial susceptibility testing

## Abstract

*Lactococcus* spp. is typically thought to be of low virulence and seldom considered pathogenic. Few cases of significant infections in children have been reported, all outside of the United States. There is also limited data on antimicrobial susceptibility testing for *Lactococcus* spp. We present three pediatric patients with central line bloodstream infections due to *Lactococcus* spp. between 2018 and 2020, along with a review of the pediatric literature.

Central venous catheters (CVC) are often essential in the pediatric population. A serious complication of central access is central line-associated blood stream infection (CLABSI), which is associated with morbidity, mortality, and financial burden. In the pediatric population, neonates, oncology, and intestinal failure patients are at greatest risk for CLABSIs (1). These infections are caused by a wide variety of pathogens that often vary based on patients' comorbidities. Historically, *Lactococcus* species have seldom been considered pathogenic in humans. However, cases of clinically significant *Lactococcus* infections have been reported (2–11). Few pediatric cases have been reported, all of which are from geographic regions outside the United States. In this case series, we present three pediatric *Lactococcus* bacteremia cases from 2018 to 2020, all thought to be CLABSI. Informed consent was not obtained for this series of case reports, as patient identifiers are not included and their anonymity and confidentiality are protected.

## Case 1

A 203 day-old, 25-week gestational age infant, during his birth admission in the neonatal intensive care unit, developed a fever to 38.8 degrees Celsius. His medical conditions included bronchopulmonary dysplasia and an ileostomy due to intestinal perforation, requiring parenteral nutrition through his CVC. The remainder of his vitals were normal, and his physical examination was unrevealing. Central and peripheral blood cultures were obtained. He was started on empiric vancomycin and piperacillin-tazobactam therapy. After 8 h of incubation, his central blood culture grew gram-positive cocci in pairs and chains as well as gram-positive bacilli. The patient's CVC was removed. Three days after initial growth, two organisms were identified: *Lactococcus lactis* spp. *lactis* and *Lactobacillus* spp. His initial and three subsequent peripheral blood cultures remained negative.

When the species were identified, the patient's antibiotic therapy was de-escalated to vancomycin monotherapy and the patient completed 10 days from first negative blood culture. Antimicrobial susceptibility testing was performed at a reference laboratory. These resulted 2 days after completion of his treatment, revealing susceptibility to ceftriaxone [MIC (minimum inhibitory concentration) 0.5 mcg/mL], penicillin (MIC 0.5 mcg/mL) and vancomycin (MIC <0.5 mcg/mL).

## Case 2

A 21-month-old term male with congenital nephrotic syndrome, stage 2 chronic kidney disease, and hypogammaglobulinemia with a CVC since 15 months of age, presented to the nephrology clinic for his biweekly albumin and furosemide infusions. During the albumin infusion, he became febrile to 38.5 degrees Celsius. He was subsequently admitted to rule out a CLABSI. Apart from chronic cough and congestion, the child was well-appearing with no alternate focus of infection.

Central and peripheral blood cultures were obtained prior to initiating empiric antibiotic therapy with cefepime. A respiratory viral panel was also obtained, which detected rhinovirus/enterovirus and adenovirus. After 14 h of incubation, the central blood culture grew gram-positive cocci in pairs and chains, at which point vancomycin was added to the antibiotic regimen. The organism was subsequently identified as *Lactococcus lactis* and was sent to a reference laboratory for antimicrobial susceptibility testing.

The peripheral blood culture obtained prior to antibiotics, as well as two repeat central blood cultures post antibiotics revealed no growth. The CVC was retained. Antimicrobial susceptibility results returned on day 8 of therapy, revealing susceptibility to ceftriaxone (MIC 0.5 mcg/mL), penicillin (MIC 0.5 mcg/mL), and vancomycin (MIC 0.25 mcg/mL). Antibiotic therapy was de-escalated to ceftriaxone monotherapy. He completed a total of 10 days of antibiotic therapy from first negative blood culture.

## Case 3

A 2-year-old male with a history of Hirschsprung's Disease and short bowel syndrome presented to an outside facility with fever and headache for 1 day. He was dependent upon Gastric-tube feedings and total parenteral nutrition, for which he had a CVC. Four weeks prior to presentation, his CVC had been repaired due to a leak. At the outside facility, his CVC was found to be occluded, and he was transferred to our hospital. On admission, physical exam was remarkable for a febrile (39.4 degrees Celsius), ill-appearing child with tachypnea, rhinorrhea, cough, and rales bilaterally. Crusting was noted adjacent to his ostomy site. An abdominal ultrasound was unrevealing.

A peripheral blood culture was drawn by the outside facility, after which he received a dose of cefepime. After alteplase was administered at our facility, a central line blood culture was obtained and patient was started on cefepime, vancomycin, metronidazole, and fluconazole. On day 2, the peripheral blood culture from the outside facility grew gram-positive cocci in chains. On day 3, the organism was identified as *Enterococcus spp*. On day 4, it was discovered that the isolate was misidentified, and was confirmed to be *Lactococcus garvieae*. Antimicrobial therapy was subsequently de-escalated to vancomycin monotherapy. An echocardiogram was performed and was negative for intracardiac vegetations. The isolate was sent to a reference laboratory for antimicrobial susceptibility testing and resulted on day 8 of admission as susceptible to ceftriaxone (MIC 0.5 mcg/mL), penicillin (MIC 1 mcg/mL), and vancomycin (MIC 0.5 mcg/mL), at which point therapy was further narrowed to ceftriaxone. Repeat blood cultures (two central and one peripheral) remained negative. The CVC was retained. He completed a total of 14 days of antibiotic therapy from first negative blood culture.

## Discussion

*Lactococcus* is a gram-positive coccus, facultative anaerobe (12) consisting of eight species (8). Previously, *Lactococcus* was classified as *Streptococcus*, and often is misidentified as *Enterococcus* (12), as demonstrated by the third case. *L. garvieae* is commonly known to cause disease in farmed fish (12), and *L. lactis* is often used as an additive in food products (3). *Lactococcus* spp. has also been documented as being part of human intestinal normal flora (3, 4). Despite *Lactococcus* spp. historically being considered non-pathogenic, there have been reports of infections in adult and pediatric patients due to *Lactococcus* species in recent years.

*Lactococcus* species, like other organisms typically regarded as contaminants or non-pathogenic, have the potential to cause clinical infection in children at risk, including those with CVCs. In this case series, we report three pediatric cases with *Lactococcus* bacteremia, all CLABSI. The terminology used to define intravascular catheter-related infections, CLABSI and catheter-related bloodstream infection (CRBSI) are used interchangeably, although their meanings differ. CLABSI is a surveillance definition used to identify a primary bloodstream infection that is unrelated to an infection at another site in a patient with a CVC. CRBSI is a clinical definition that requires fulfillment of criteria in order to attribute the catheter as the source of the bloodstream infection (13). Confirming CRBSI in the pediatric population is challenging, as collecting peripheral blood cultures with sufficient volume of blood can be difficult (1) and colony-forming unit (CFU) and quantitative cultures of catheter-tip, central and peripheral blood cultures are seldom performed. Although *Lactococcus* species were not isolated from both central and peripheral cultures in our patients, we believe all infants had CLABSI due to rapid growth, fever at the time of infusion and/or absence of alternative explanation for fever. Per the National Healthcare Safety Network (NHSN), all our *Lactococcus* species cases met criteria for CLABSI-I, none of which fulfilled mucosal barrier injury-I criteria (14). Cases 1 and 2 were present on admission. Case 3 was a healthcare associated infection and was reported to NHSN.

We reviewed the literature of pediatric infections secondary to *Lactococcus* species and found 11 additional cases (Table 1). There are a greater number of reported pediatric infections due to *L. lactis* compared to *L. garvieae* (1, 8). The most common source of infection is blood (45%), with 60% (three of five) occurring in patients with central lines. Other sources include liver abscess (9%), brain abscess (9%), cerebrospinal fluid (9%), urine (9%), and endocarditis (18%). Median patient age was 12 months (range, 14 days−14 years). Six of 11 patients (54%) had an underlying risk factor. Duration of therapy ranged from 7 to 40 days depending on the source of infection. Most definitive treatment regimens consisted of a third-generation cephalosporin (45%). Of BSIs, 80% (four of five) of infants received vancomycin as part of their definitive therapy.

**Table 1:** Cases of *Lactococcus* spp. infections in pediatric patients (<18 years of age) in English, peer-reviewed literature since 2000 in PubMed database.

**References**	**Year, location**	**Age**	**Possible risk factors**	**Species**	**Source**	**CVC removed**	**Antimicrobial therapy; treatment duration (*d*)**	Susceptibility interpretation and MICs^*^(mcg/mL unless specified)
Nakarai et al. (6)	2000, Japan	14 years	None	*Lactococcus lactis*	Liver abscess	N/A	CTM, AMK, CLN, PAN, PIP; 35	• 3+: PIP, CTM, ERY, CLN, MIN, PAN • 1+: AMK
Glikman et al. (2)	2010, Israel	9 months	• SBS • TPN • CVC	*L. lactis*	Blood (central and peripheral sources)	No	CFO, VAN; 10	S: PEN (0.25), CEF (0.125), VAN (0.5)
Topcu et al. (8)	2011, Turkey	19 months	Ingestion of raw milk 13 months prior	*L. lactis*	Brain abscess	N/A	CEF, VAN, MER; 40	Not reported
Uchida et al. (9)	2011, Japan	16 days	Prematurity (36 weeks GA)	*L. lactis*	CSF	N/A	CFO; 12	S: AMP, PEN, CFO, CFP, ERY, CLA, CLN, VAN, LEV, CHL, MER
Newby et al. (7)	2014, Canada	14 days	• Prematurity (31 weeks GA) • Contaminated maternal breast milk (*L. lactis*)	*L. lactis*	Urine	N/A	CFO; 7	• S: CFO, VAN, MER, AMO-CLA • I: PEN, CEP • R: CFX, AMO
Karaaslan et al. (3)	2014, Turkey	1 year	• Down syndrome Hirschsprung's Disease TPN • CVC	*L. lactis*	Blood (central and peripheral)	No	VAN; 10	S: VAN
Mitra N, et al. (5)	2015, India	3 years	Gingivitis, Stomatitis, R cervical lymphadenitis, Fish and unpasteurized milk intake	*Lactococcus garvieae*	Blood	N/A	AMO; not reported	S: AMO
Taniguchi et al. (10)	2015, Japan	4 months	None	*L. lactis*	Endocarditis	N/A	None (patient deceased)	Not reported
Karaaslan et al. (11)	2016, Turkey	5 months	Ileostomy	*L. lactis*	Blood (peripheral)	N/A	VAN; 24	• S: VAN (0.5) • I: PEN (0.5)
Karaaslan et al. (11)	2016, Turkey	6 months	• TPN • CVC	*L. lactis*	Blood (central)	Yes	VAN; 10	S: VAN (0.5), PEN
Mansour et al. (4)	2016, Israel	11 years	None	*L. lactis*	Endocarditis	N/A	CFA, CEF; 29	Not reported
Presented Case #1	2018, United States	6 months	• SBS • TPN • CVC	*L. lactis*	Blood (central)	Yes	VAN; 10	S: PEN (0.5), CEF (0.5), VAN (0.5):
Presented Case #2	2020, United States	21 months	• Congenital nephrotic syndrome • Stage II CKD • Hypogammaglobulinemia • CVC	*L. lactis*	Blood (central)	No	CEF; 10	S: PEN (0.5), CEF (0.5), VAN (0.25)
Presented Case #3	2020, United States	2 years	• SBS • Hirschsprung's Disease TPN • CVC	*L. garvieae*	Blood (peripheral)	No	CEF; 14	S: PEN (1), CEF (0.5), VAN (0.5)

* *If reported*.

*AMK, amikacin; AMO, amoxicillin; AMO/CLA, amoxicillin-clavulanate; AMP, ampicillin; CEF, ceftriaxone; CEP, cephalexin; CFA, cefazolin; CFO, cefotaxime; CFP, cefepime; CFX, cefixime; CHL, chloramphenicol; CKD, chronic kidney disease; CLA, clarithromycin; CLN, clindamycin; CSF, cerebrospinal fluid; CTM, cefotiam; CVC, central venous catheter; ERY, erythromycin; GA, gestational age; I, intermediate; LEV, levofloxacin; MER, meropenem; MIN, minocycline; PAN, panipenem; PEN, penicillin; PIP, piperacillin; R, resistant; S, susceptible; SBS, short bowel syndrome; TPN, total parenteral nutrition; VAN, vancomycin*.

To the best of our knowledge, this series highlights the first reported pediatric cases of *Lactococcus* species infections in the United States. Our pediatric cases add to the scarce pool of existing data. All of our patients were in the infant-toddler age group, which is similar to international pediatric cases of *Lactococcus* BSIs (Table 1). The definitive source of *Lactococcus* bacteremia in these patients is not known. None of our patients were receiving probiotics. Two of our 3 cases did not take oral feedings, making dietary risk factors unlikely. Similar to the reported cases in Table 1 without known dietary risk factors, 2 of the 3 BSIs occurred in children with short bowel syndrome and all had the presence of a CVC, suggesting that bacterial translocation may be an inciting factor for these cases of CLABSI. *Lactococcus* species have been isolated from the gastrointestinal tract and may be a commensal organism that is part of the normal flora (4). One of our patients (Case 2) had a known immunodeficiency of hypogammaglobulinemia. However, none of the reported cases in Table 1 noted an immunodeficiency, suggesting that immune status is not a contributing factor to *Lactococcus* infections. Two of our 3 patients were treated for a total duration of 10 days, and two of our three patients were managed with CVC retention without recurrence of infection. This is consistent with the management of pediatric *Lactococcus* bloodstream infections in the literature (Table 1).

Isolates were identified using mass spectrometry. Antimicrobial susceptibility was performed by gradient diffusion and interpreted according to The Clinical Laboratory Standards Institute (CLSI) guidelines. CLSI has published susceptibility breakpoints for *Lactococcus* spp., including penicillin (MIC ≤ 1), ceftriaxone (MIC ≤ 1), and vancomycin (MIC ≤ 2) (15). Susceptibility testing for this organism is typically performed at reference laboratories, which can have a longer turnaround time (7–12 days for our reported cases). Seven of 11 previously published cases reported quantitative antimicrobial sensitivity testing (AST) to beta-lactam antibiotics, of which 6 (85%) were susceptible (Table 1). All three of our reported cases were susceptible to penicillin, ceftriaxone, and vancomycin, adding further AST data to the limited literature. The continued accrual of antimicrobial susceptibility data may bring forth the possibility of empirically treating *Lactococcus* spp. bacteremia with a 3rd generation cephalosporin.

In conclusion, we report three pediatric *Lactococcus* spp. CLABSI, two of which were successfully treated with CVC retention. These cases add to the limited literature regarding this organism—previously thought to be of low pathogenicity in humans—and its AST, suggesting that *Lactococcus* spp. should be considered pathogenic in the appropriate circumstances.

## Data Availability Statement

The original contributions presented in the study are included in the article/supplementary material, further inquiries can be directed to the corresponding author/s.

## Author Contributions

SF, AB, and HM conceptualized and designed the study, drafted the initial manuscript, and reviewed and revised the manuscript. All authors approved the final manuscript as submitted and agree to be accountable for all aspects of the work.

## Funding

Funding for open access publishing was provided by Departmental Cabe Funding of the University of Arkansas for Medical Sciences.

## Conflict of Interest

The authors declare that the research was conducted in the absence of any commercial or financial relationships that could be construed as a potential conflict of interest.

## Publisher's Note

All claims expressed in this article are solely those of the authors and do not necessarily represent those of their affiliated organizations, or those of the publisher, the editors and the reviewers. Any product that may be evaluated in this article, or claim that may be made by its manufacturer, is not guaranteed or endorsed by the publisher.
